# Supplementary material for: Three distinct developmental pathways for adaptive and two IFN-γ-producing γδ T subsets in adult thymus
Source: Nat Commun. 2017 Dec 4;8:1911. doi: 10.1038/s41467-017-01963-w (PMC5715069; doi:10.1038/s41467-017-01963-w)
Supplement: Supplementary file 1 — Supplementary Information [file 41467_2017_1963_MOESM1_ESM.pdf]

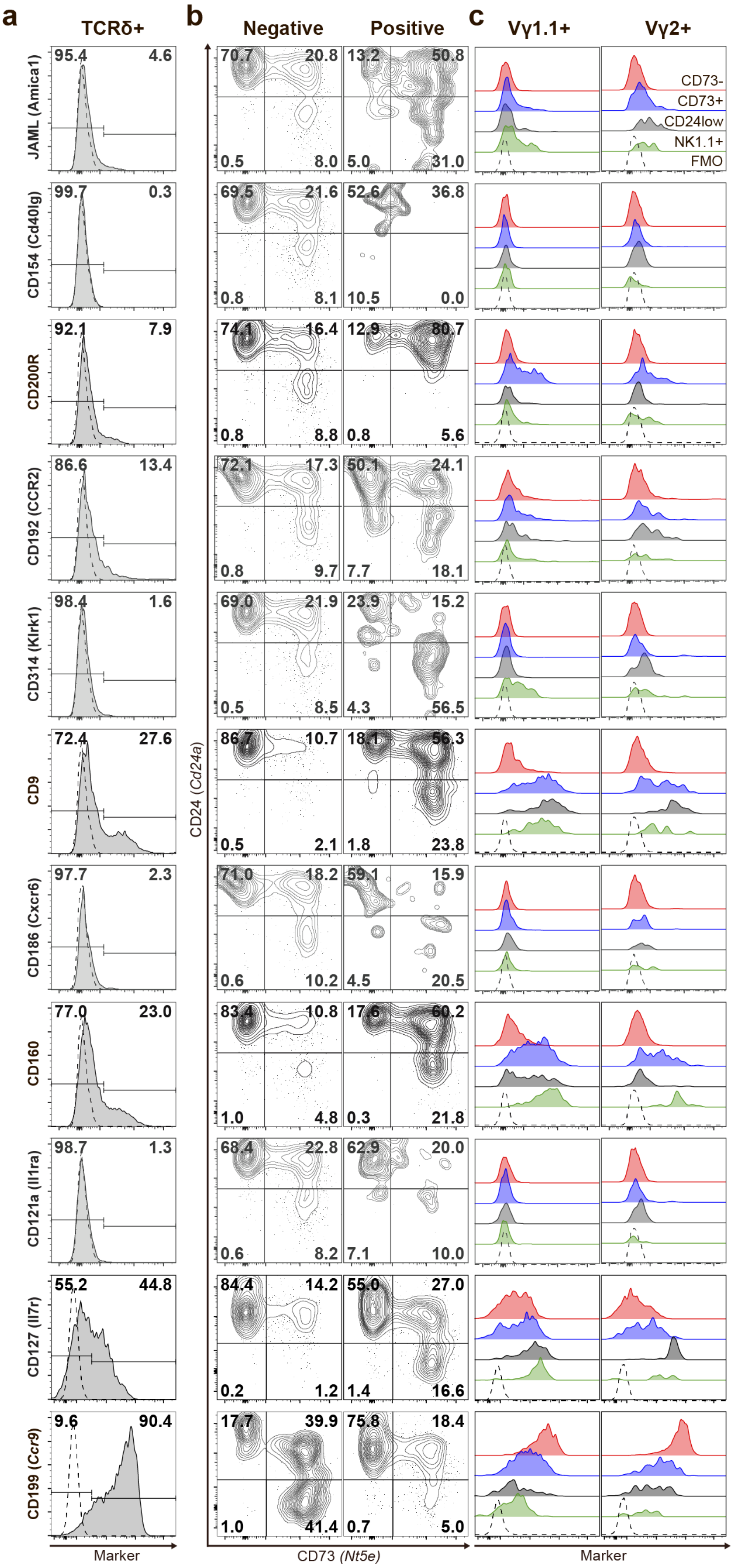

Supplementary Figure 1: Expression of surface markers during γδ T cell development

Representative flow cytometric plots showing the expression of the putative surface markers identified in Fig. 1b. Plots show (**a**) TCRδ^{+} gated thymocytes as histograms, (**b**) the distribution of marker-negative and marker-positive cells within developmental stages defined by CD73 and CD24 and (**c**) the expression of markers within subpopulations of the Vγ1.1^{+} and the Vγ2^{+} subset.

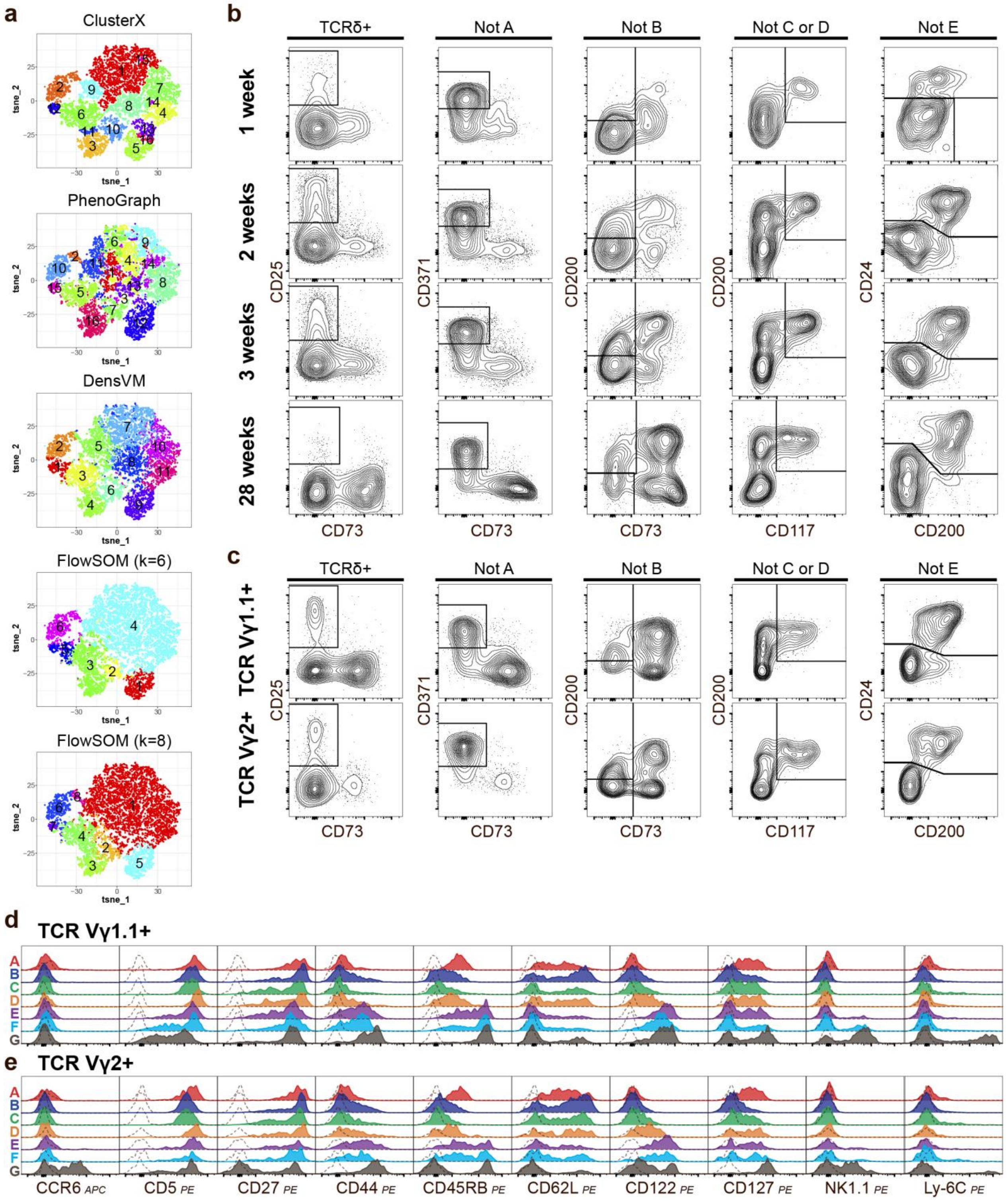

Supplementary Figure 2: Surface expression of CD117, CD200 and CD371 allows isolation of seven distinct γδ thymocyte populations

(**a**) Clustering of TCRδ$^+$ thymocytes based on the expression of CD24, CD25, CD73, CD117, CD200 and CD371 visualised by t-Distributed Stochastic Neighbour Embedding (t-SNE) and clustered by different automated clustering algorithms included in the "cytofkit" R package: ClusterX, PhenoGraph, DensVM and FlowSOM (with k set to 6 or 8).

(**b**) Population A to G gating of TCRδ$^+$ thymocytes from 1- to 28-week-old mice.

(**c**) Population A to G gating of TCRδ$^+$ Vγ1.1$^+$ (top) and Vγ2$^+$ (bottom) cells of 8-week-old mice.

(**d,e**) Representative histograms of previously established surface markers of γδ T cells within populations A to G divided into the (**d**) Vγ1.1$^+$ and (**e**) Vγ2$^+$ subset (n = 6 mice from two independent experiments).

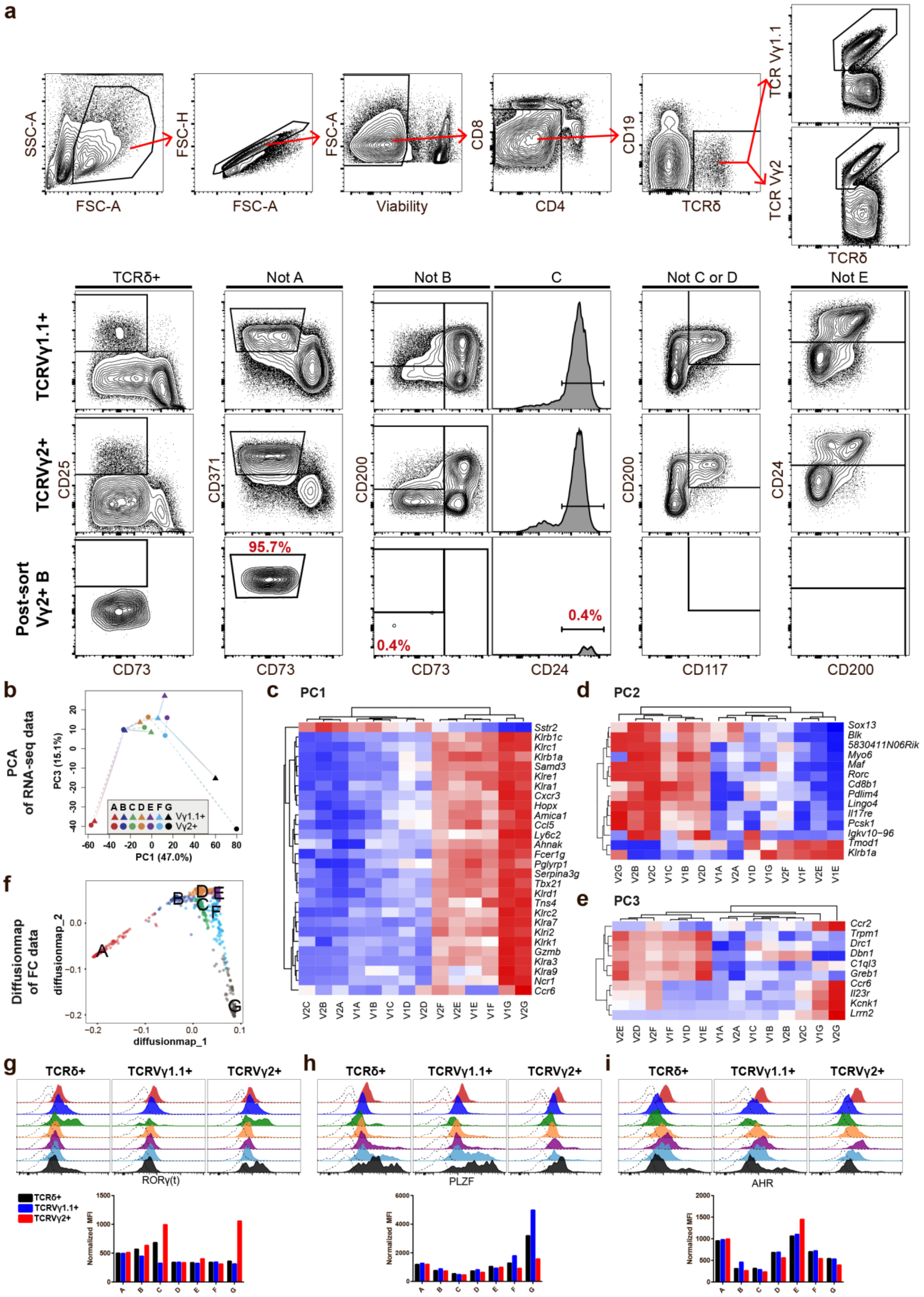

a
SSC-A
FSC-A
FSC-H
FSC-A
Viability
CD8
CD4
CD19
TCRδ
TCR Vγ1.1
TCR Vγ2
TCRδ
TCRδ+
Not A
Not B
C
Not C or D
Not E
TCRVγ1.1+
TCRVγ2+
Post-sort Vγ2+ B
CD25
CD371
CD200
CD200
CD24
95.7%
0.4%
0.4%
CD73
CD73
CD73
CD24
CD117
CD200
b
PCA of RNA-seq data
PC3 (15.1%)
PC1 (47.0%)
A B C D E F G
Vγ1.1+
Vγ2+
c
PC1
d
PC2
e
PC3
f
Diffusionmap of FC data
diffusionmap_2
diffusionmap_1
g
h
i
RORγ(t)
PLZF
AHR
TCRδ+
TCRVγ1.1+
TCRVγ2+
Normalized MFI

Supplementary Figure 3: Populations A to G can be sorted and exhibit differential expression

(**a**) Gating strategy for FACS sorting of populations A to G from the Vγ1.1$^+$ and the Vγ2$^+$ subsets and post-sorting purity from the sorted Vγ2$^+$ B population.

(**b-e**) Principal component analysis and heat maps showing expression of genes with numeric loadings greater than 0.05 (indicating the genes most important for separation of the populations) within the (**c**) principal component 1 (PC1), (**d**) PC2 and (**e**) PC3.

(**f**) Cellular progression predicted by flow cytometric expression of CD24, CD25, CD73, CD117, CD200 and CD371 by diffusion map. Each A to G population was reduced to 100 cells before analysis. Each dot represents a single cell.

(**g-i**) Expression effector subset-related transcription factors assayed by flow cytometry and visualized by histograms and the normalized geometric mean fluorescence intensity (MFI) of (**f**) RORγ(t), (**g**) PLZF and (**h**) AHR. Dashed lines depict the signal from isotype controls within the same gates.

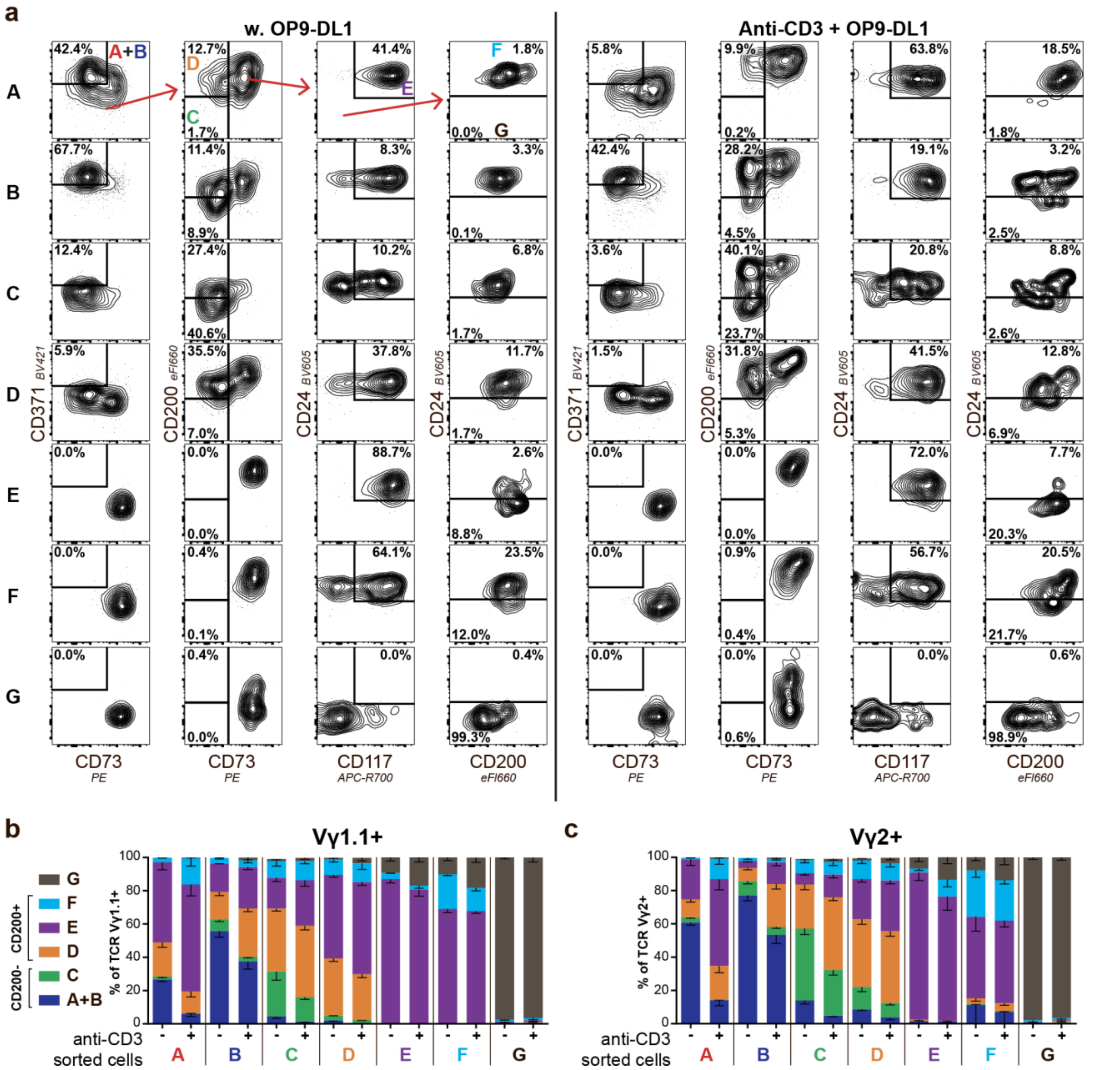

Supplementary Figure 4: Progression through the D, E and F populations is induced by TCR signalling

(**a**) Representative flow cytometry plots of sorted cells divided into the A to G populations after two days of culture on OP9-DL1 monolayers in the presence (anti-CD3 + OP9-DL1) or absence (w. OP9-DL1) of anti-CD3. Note that the E population is gated as CD24^{+}CD117^{+} to avoid misclassifying E cells in the CD24^{-} population (not observed *in vivo*).

(**b,c**) Progression of sorted TCRδ^{+} cells from population A to G after two days of culture on OP9-DL1 monolayers in the presence or absence of immobilized anti-CD3. Quantified as the percent of (**b**) Vγ1.1^{+} and (**c**) Vγ2^{+} cells. Bars depict the mean ± SEM from three independent experiments with cells sorted from four to eight mice.

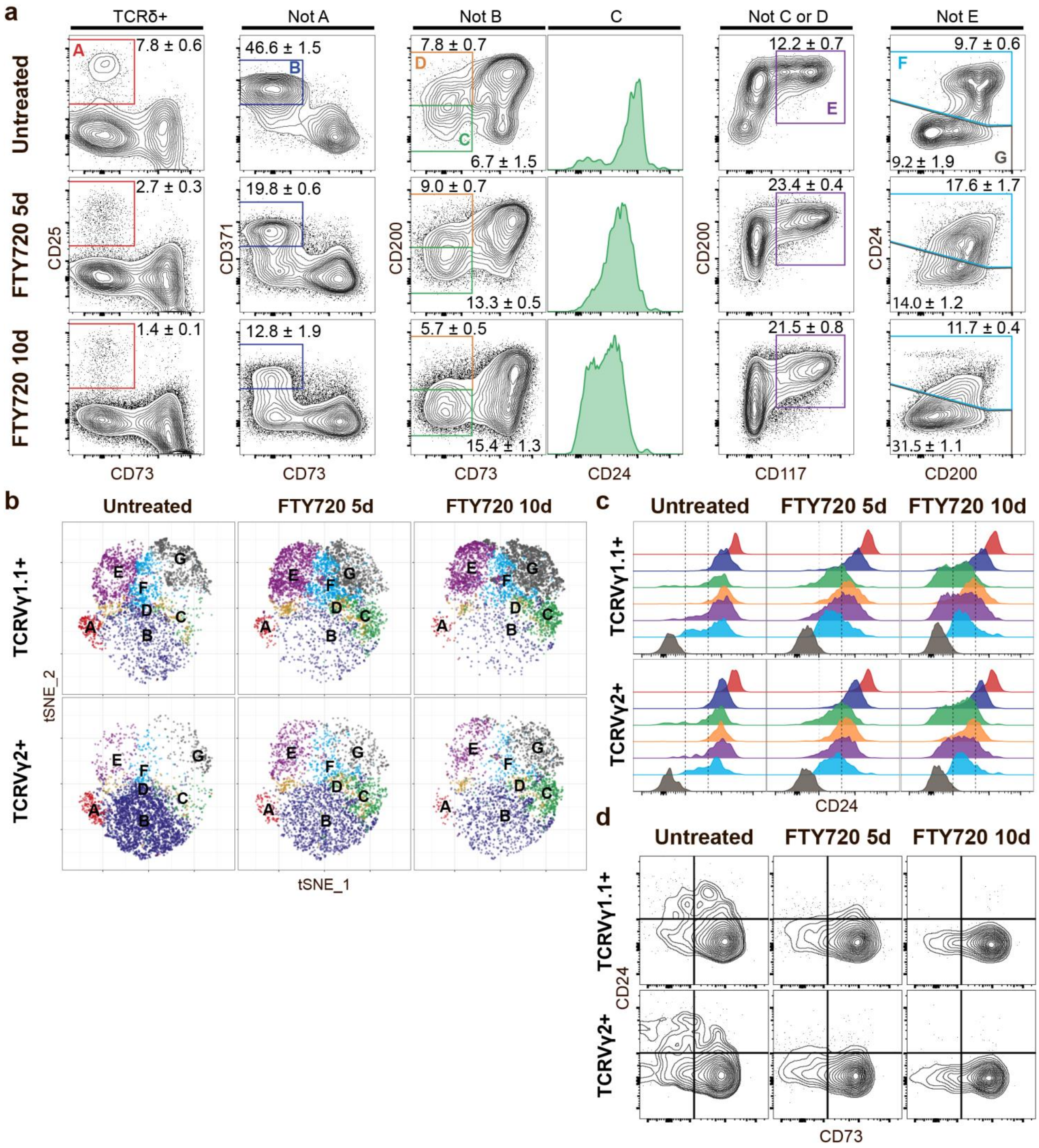

Supplementary Figure 5: γδ T cells emigrate from the thymus at the C, E and G stages

(**a,b**) TCRδ$^+$ thymocyte distributions within population A to G after treatment with FTY720 for 5 or 10 days visualized by (**a**) standard bi-axial flow cytometry plots of the gating strategy and (**b**) t-SNE map of population A to G of the Vγ1.1$^+$ and Vγ2$^+$ subsets. Numbers denote the mean ± SEM.

(**c**) Representative histograms of CD24 expression within the A to G populations of the Vγ1.1$^+$ and Vγ2$^+$ subsets after treatment with FTY720 for 5 or 10 days.

(**d**) Representative bi-axial plots showing expression of CD24 within CD73$^-$ and CD73$^+$ TCRδ$^+$ Vγ1.1$^+$ and Vγ2$^+$ cells from inguinal lymph nodes after FTY720 treatment for 5 or 10 days.

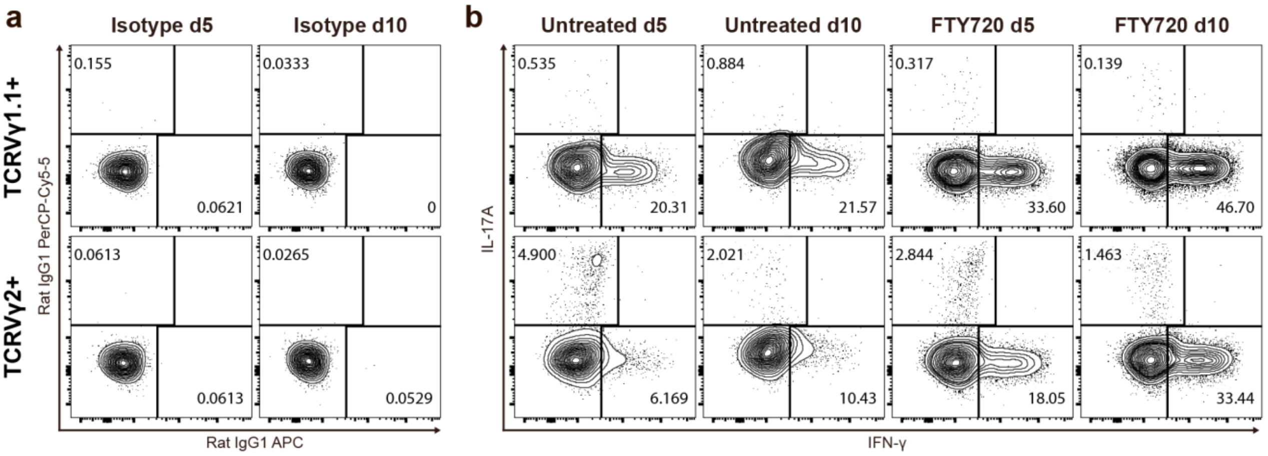

Supplementary Figure 6: IFN-γ-, but not IL-17A-, producing cells accumulate when thymic export is inhibited

Representative flow cytometry plots showing (**a**) isotype fluorescence signal and (**b**) the expression of IFN-γ and IL-17A within the Vγ1.1^{+} and Vγ2^{+} subsets after treatment with FTY720 for 5 or 10 days.

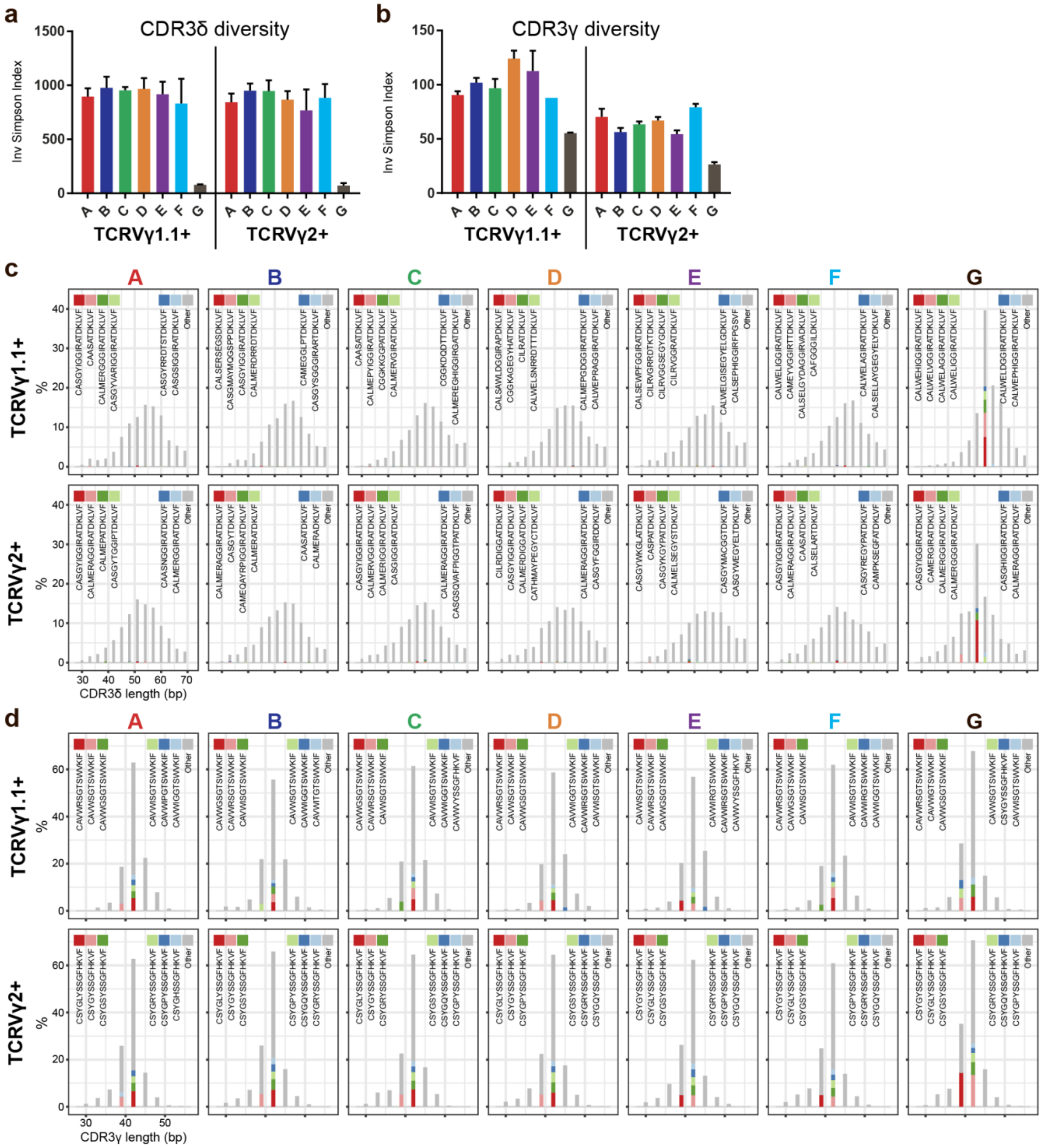

Supplementary Figure 7: The G populations exhibit low TCR diversity

(**a,b**) Complementarity determining region 3 (CDR3) clonotype diversity calculated by inverse Simpson Indices from RNA-Seq of the A to G populations of (**a**) CDR3δ and (**b**) CDR3γ.

(**c,d**) Stacked bar plots of (**c**) CDR3δ and (**d**) CDR3γ showing the frequency (y-axis) of the six most frequent clonotypes within the A to G populations of Vγ1.1^{+} and Vγ2^{+} distributed according to the CDR3 nucleotide length (x-axis).

Supplementary Table 1: Antibodies and reagents

REAGENT	DILUTION	SOURCE	IDENTIFIER
**Antibodies**			
CD3 *(145-2C11)*	1:200	BioLegend	Cat# 100340
CD3 *(145-2C11)* BV786	1:200	BD Biosciences	Cat# 564379
CD4 *(RM4-5)* BV650	1:200	BD Biosciences	Cat# 563747
CD4 *(GK1.5)* BUV737	1:200	BD Biosciences	Cat# 564298
CD5 *(53-7.3)* PE	1:400	BD Biosciences	Cat# 553023
CD8 *(53-6.7)* BV711	1:200	BD Biosciences	Cat# 563046
CD8 *(53-6.7)* BV650	1:200	BioLegend	Cat# 100742
CD8 *(53-6.7)* BUV395	1:200	BD Biosciences	Cat# 563786
CD9 *(MZ3)* PE	1:50	BioLegend	Cat# 124805
CD19 *(1D3)* BV650	1:200	BD Biosciences	Cat# 563235
CD24 *(M1/69)* BV421	1:200	BD Biosciences	Cat# 562563
CD24 *(M1/69)* BV510	1:200	BD Biosciences	Cat# 563115
CD24 *(M1/69)* BV605	1:200	BD Biosciences	Cat# 563060
CD24 *(M1/69)* AlexaFluor700	1:200	BD Biosciences	Cat# 564237
CD25 *(PC61)* BV786	1:200	BD Biosciences	Cat# 564023
CD25 *(PC61)* AlexaFluor700	1:200	BioLegend	Cat# 102024
CD25 *(PC61)* APC-Cy7	1:200	BioLegend	Cat# 102026
CD27 *(LG.3A10)* PE	1:200	BioLegend	Cat# 124210
CD44 *(IM7)* PE	1:800	BD Biosciences	Cat# 553134
CD45RB *(C363-16A)* BV421	1:200	BD Biosciences	Cat# 562849
CD45RB *(C363-16A)* PE	1:6400	BioLegend	Cat# 103307
CD62L *(MEL-14)* PE	1:6400	BD Biosciences	Cat# 553151
CD73 *(TY/11.8)* PerCP-eFluor710	1:200	eBioscience	Cat# 46-0731
CD73 *(TY/11.8)* APC	1:200	BioLegend	Cat# 127210
CD73 *(TY/11.8)* BV605	1:50	BioLegend	Cat# 127215
CD117 *(2B8)* BV421	1:50	BioLegend	Cat# 105827
CD117 *(2B8)* APC-R700	1:50	BD Biosciences	Cat# 565476
CD121a *(JAMA-147)* PE	1:50	BioLegend	Cat# 113505
CD122 *(TM-β1)* PE	1:200	BioLegend	Cat# 123209
CD127 *(A7R34)* BV421	1:50	BioLegend	Cat# 135024
CD127 (*A7R34*) PE	1:200	BioLegend	Cat# 135009
CD154 *(MR1)* PE	1:50	BD Biosciences	Cat# 553658
CD160 *(7H1)* PE	1:50	BioLegend	Cat# 143004
CD186 *(221002)* PE	1:50	R&D Systems	Cat# FAB2145P
CD192 *(475301)* PE	1:50	R&D Systems	Cat# FAB5538P
CD196 *(29-2L17)* APC	1:25	BioLegend	Cat# 129813
CD199 *(9B1)* PE	1:50	BioLegend	Cat# 129707
CD200 *(OX-90)* eFluor660	1:50	eBioscience	Cat# 50-5200
CD200 *(OX-90)* Biotin	1:50	ThermoFisher	Cat# MA5-17979
CD200 (*OX-90*) BV421	1:50	BD Biosciences	Cat# 565547
CD200R *(OX-110)* PE	1:50	BioLegend	Cat# 123907
CD314 *(C7)* PE	1:50	eBioscience	Cat# 12-5882
CD371 *(5D3/CLEC12A)* PE	1:50	BioLegend	Cat# 143403
CD371 *(5D3/CLEC12A)* BV421	1:50	BD Biosciences	Cat# 564795
JAML *(4E10)* PE	1:50	BioLegend	Cat# 128503
Ly-6C *(HK1.4)* PE	1;400	BioLegend	Cat# 128008
TCRδ *(GL-3)* PE-CF594	1:200	BD Biosciences	Cat# 563532
TCRβ *(H57-597)* BV711	1:200	BD Biosciences	Cat# 563135
TCRVγ1.1 *(2.11)* FITC	1:100	BioLegend	Cat# 141104
TCRVγ1.1 *(2.11)* PE	1:200	BioLegend	Cat# 141106
TCRVγ2 *(UC3-10A6)* PE-Cy7	1:200	eBioscience	Cat# 25-5828
TCRVδ6.3/2 *(8F4H7B7)* PE	1:50	BD Biosciences	Cat# 555321
TCRVδ4 *(GL2)* eFluor660	1:50	eBioscience	Cat# 50-5702
NK1.1 *(PK136)* BV650	1:50	BioLegend	Cat# 108736
NK1.1 (PK136) PE	1:50	BD Biosciences	Cat# 553165
IL-17A *(TC11-18H10)* PerCP-Cy5.5	1:50	BioLegend	Cat# 506920
IFN-γ *(XMG1.2)* PE	1:50	BD Biosciences	Cat# 554412
IFN-γ *(XMG1.2)* APC	1:50	BioLegend	Cat# 505810
PLZF *(9E12)* PE	1:50	BioLegend	Cat# 145803
AHR *(T49-550)* PE	1:50	BD Biosciences	Cat# 565711
RORγ(t) *(B2D)* PE	1:50	eBioscience	Cat# 12-6981
Rat IgG1 isotype *(RTK2071)* PerCP-Cy5.5	1:50	BioLegend	Cat# 400426
Rat IgG1 isotype *(R3-34)* PE	1:50	BD Biosciences	Cat# 554685
Rat IgG1 isotype *(RTK2071)* APC	1:50	BioLegend	Cat# 400412
**Chemicals, Peptides, and Recombinant Proteins**			
Fixable Viability Dye eFluor506	1:100	eBioscience	Cat# 65-0866
Fixable Viability Dye eFluor780	1:800	eBioscience	Cat# 65-0865
7-AAD Viability Staining Solution	5:400	BioLegend	Cat# 420404
Streptavidin BV510	1:200	BD Biosciences	Cat# 563261
FTY720		Sigma-Aldrich	Cat# SML0700
TRI Reagent		Sigma-Aldrich	Cat# 93289
1-Bromo-3-chloropropane (BCP)		Sigma-Aldrich	Cat# B9673
Brilliant Stain Buffer		BD Biosciences	Cat# 563794
Glutamax MEM-α		Gibco	Cat# 32561-029
